# Microbial Natural Products with Antiviral Activities, Including Anti-SARS-CoV-2: A Review

**DOI:** 10.3390/molecules27134305

**Published:** 2022-07-05

**Authors:** Andri Frediansyah, Fajar Sofyantoro, Saad Alhumaid, Abbas Al Mutair, Hawra Albayat, Hayyan I. Altaweil, Hani M. Al-Afghani, Abdullah A. AlRamadhan, Mariam R. AlGhazal, Safaa A. Turkistani, Abdulmonem A. Abuzaid, Ali A. Rabaan

**Affiliations:** 1 PRTPP, National Research and Innovation Agency (BRIN), Yogyakarta 55861, Indonesia; 2 Faculty of Biology, Gadjah Mada University, Yogyakarta 55281, Indonesia; fajar.sofyantoro@ugm.ac.id; 3 Administration of Pharmaceutical Care, Al-Ahsa Health Cluster, Ministry of Health, Al-Ahsa 31982, Saudi Arabia; saalhumaid@moh.gov.sa; 4 Research Center, Almoosa Specialist Hospital, Al-Ahsa 36342, Saudi Arabia; abbas.almutair@almoosahospital.com.sa; 5 College of Nursing, Princess Norah Bint Abdulrahman University, Riyadh 11564, Saudi Arabia; 6 School of Nursing, Wollongong University, Wollongong, NSW 2522, Australia; 7 Nursing Department, Prince Sultan Military College of Health Sciences, Dhahran 33048, Saudi Arabia; 8 Infectious Disease Department, King Saud Medical City, Riyadh 7790, Saudi Arabia; hhalbayat@gmail.com; 9 Department of Clinical Laboratory Sciences, Mohammed Al-Mana College of Health Sciences, Dammam 34222, Saudi Arabia; hayyana@nachs.edu.sa; 10 Laboratory Department, Security Forces Hospital, Makkah 24269, Saudi Arabia; hmalafghani@sfhm.med.sa; 11 Gene Center for Research and Training, Jeddah 2022, Saudi Arabia; 12 Laboratory and Toxicology Department, Security Forces Specialized Comprehensive Clinics, Al-Ahsa 36441, Saudi Arabia; aramadhan@moimsd.gov.sa; 13 Hematopathology Department, Dammam Regional Laboratory, Dammam 1854, Saudi Arabia; dr.m.r.alghazal@hotmail.com; 14 Fakeeh College for Medical Sciences, Jeddah 21134, Saudi Arabia; turkistanisafaa1@gmail.com; 15 Medical Microbiology Department, Security Forces Hospital Programme, Dammam 32314, Saudi Arabia; monem997@yahoo.com; 16 Molecular Diagnostic Laboratory, Johns Hopkins Aramco Healthcare, Dhahran 31311, Saudi Arabia; 17 College of Medicine, Alfaisal University, Riyadh 11533, Saudi Arabia; 18 Department of Public Health and Nutrition, Faculty of Basic and Applied Sciences, University of Haripur, Haripur 22610, Pakistan

**Keywords:** natural products, microorganism, SARS-CoV-2, COVID-19, aurasperone, aspulvinone

## Abstract

The SARS-CoV-2 virus, which caused the COVID-19 infection, was discovered two and a half years ago. It caused a global pandemic, resulting in millions of deaths and substantial damage to the worldwide economy. Currently, only a few vaccines and antiviral drugs are available to combat SARS-CoV-2. However, there has been an increase in virus-related research, including exploring new drugs and their repurposing. Since discovering penicillin, natural products, particularly those derived from microbes, have been viewed as an abundant source of lead compounds for drug discovery. These compounds treat bacterial, fungal, parasitic, and viral infections. This review incorporates evidence from the available research publications on isolated and identified natural products derived from microbes with anti-hepatitis, anti-herpes simplex, anti-HIV, anti-influenza, anti-respiratory syncytial virus, and anti-SARS-CoV-2 properties. About 131 compounds with in vitro antiviral activity and 1 compound with both in vitro and in vivo activity have been isolated from microorganisms, and the mechanism of action for some of these compounds has been described. Recent reports have shown that natural products produced by the microbes, such as aurasperone A, neochinulin A and B, and aspulvinone D, M, and R, have potent in vitro anti-SARS-CoV-2 activity, targeting the main protease (M^pro^). In the near and distant future, these molecules could be used to develop antiviral drugs for treating infections and preventing the spread of disease.

## 1. Introduction

The resurgence and re-emergence of fatal viral infections pose a grave threat to public health. The emergence and spread of animal viruses are existential threats to humanity due to a number of intertwined and synergistic events, such as altered human behaviors [1], high-density rapid urbanization and demographic shift [2], modernization that encourages people with high mobility [3], large gatherings [4], global warming and destruction that altered the ecosystem [5,6], and an inadequate global public health system [7]. As the societies continue to expand in size and complexity, infectious agents have an ever-increasing number of opportunities to invade the ecological niches [8]. Viruses are infectious agents that pose a growing global threat to public health. There are numerous types of viruses with various particle types, such as ribonucleic acid (RNA) and deoxyribonucleic acid (DNA) forms, positive and negative sense, and some that form in partially double-stranded DNA [9]. Some of the disease-causing viruses have been studied extensively for decades, such as the human immunodeficiency virus (HIV), hepatitis virus, herpes simplex virus (HSV), respiratory syncytial virus (RSV), and influenza virus; whereas others have only recently become a public health concern, such as the novel severe acute respiratory syndrome coronavirus 2 (SARS-CoV-2), which is responsible for the coronavirus disease 2019 (COVID-19) pandemic. Up until recently, SARS-CoV-2 has infected over half a billion people and killed over six million, with a high mortality rate among the elderly and those with comorbid conditions [10].

In addition to vaccines, drugs are one of the most reliable ways and means for defusing pandemic and epidemic risks, constituting the bedrock of infectious disease outbreak management. Thus, there is an urgent need for the discovery and repurposing of new drugs, given the rapid emergence and reemergence of new diseases. In addition, the discovery of antiviral compounds increases the likelihood of additional health benefits for humans. Microbes have evolved numerous survival strategies due to the wide variety of habitats they inhabit and their need to compete against diverse organisms [11]. They hold enormous promise as a bioresource for discovering biologically active natural products including antiviral, antibacterial, antiparasitic, antifungal, anticancer, and immunosuppressive properties [12,13]. Moreover, natural products also make excellent candidates for drug discovery because their chemical diversity is more closely aligned with drugs than that of synthetic libraries, making them a rich source of pharmacologically active substances that aid in the generation of different drugs [14,15,16]. The success of microbial natural products as drug leads depends on advancements in technologies, such as source and sampling techniques [17], the advancement of nuclear magnetic resonance (NMR) for structure determination [18], fermentation and biotechnology [19], as well as synthesis [20]. Thus, the goal of this review was to provide comprehensive information by incorporating evidence from the available research publications on isolated and identified various natural products derived from microbes, structural scaffold, in vitro or/and in vivo efficacy, and new progress related to their antiviral properties. We believe it could serve as a starting point for prioritizing molecule screening or re-investigating recent viral infections, including SARS-CoV-2.

## 2. Anti-Human Immunodeficiency Virus

Human immunodeficiency virus (HIV) is a type of retrovirus that infects humans [21]. The primary transmission mode is genital-to-genital contact, blood, sperm, and blood transfusion. This virus attacks the body’s immune system, leading to acquired immunodeficiency syndrome (AIDS), a condition in which the immune system gradually fails, allowing dangerous opportunistic infections and cancer to develop. HIV primarily infects cluster of differentiation 4^+^ (CD4^+^) T cells, dendritic cells, and macrophages [22].

Furthermore, the condition may reduce the number of CD4^+^ T cells to a critical level, resulting in a loss of cell-mediated immunity and greater susceptibility to opportunistic infection, eventually leading to AIDS [23]. As of 2019, the World Health Organization (WHO) estimates that 38 billion people worldwide are infected with HIV [24]. However, approximately 1.7 million people were unaware they were HIV-positive [24]. Therefore, several antiretroviral drugs that may slow the progression of HIV in the body have been discovered and developed. Antiretroviral drugs were only recently available to 67% of the world’s population. Lopinavir, darunavir, atazanavir, and saquinavir are protease inhibitors, while lamivudine, stavudine, emtricitabine, efavirenz, nevirapine, and rand aziridine are reverse transcription inhibitors [7]. However, no HIV drug on the market can cure HIV.

Natural products produced by microorganisms, as shown in Table 1, could be used to develop anti-HIV medications. Anti-HIV bioactive compounds from fungi are widely considered to be one of the most promising sources. Several compounds, including alachalasin A from *Podospora vesticola* fungus cultures, have been identified as effective HIV-1 replication suppressors in cellosaurus cells C8166 [25,26]. The half-maximal effective concentration, or EC_50_, of alachalasin is 8.01 μM. Pestalofone A, as well as its derivatives, including pestalofone B and E, as well as pestaloficiol G, H, J, and K isolated from the *Pestalotiopsis fici* fungus, possess anti-HIV activity [27,28]. Furthermore, epicoccin G and H were isolated from ascomycete *Epicoccum nigrum* fermentation culture, in addition to its diphenylalazine A [29]. Another study discovered that bacillamide B, derived from the ascomycete *Tricladium* sp., exhibited anti-HIV activity [30]. Furthermore, cytochalasan alkaloids, such as armochaetoglobin K, L, M, N, O, P, Q, and R, purified from the arthropod-associated *Chaetomium globosum* fungus had significant anti-HIV activity (EC_50_ = 0.25–0.55 μM) [31].

An ocean-dwelling fungus is one of the most potent sources of HIV-combating compounds. Meroterpenoids with a phenylspirodrimane skeleton, such as stachybotrin D, derived from the sponge-derived fungus *Stachybotrys chartarum* MXH-X73, were able to inhibit HIV-1 replication by targeting the reverse transcriptase enzyme [32]. This fungus was discovered on the island of Xisha in China, where it was isolated from the marine sponge *Xestospongia testudinaris* [32]. Furthermore, stachybotrysams A, B, and C, extracted from a different strain of *Stachybotrys chartarum*, also showed strong HIV-inhibitory activity [33]. Another report showed that chartarutine B, G, and H, which are all derived from the sponge-associated *Stachybotrys chartarum*, have shown significant antiviral activity against the HIV-1 virus [34]. In addition, malformin C, derived from the marine fungus *Aspergillus niger* SCSIO Jcsw6F30, demonstrated significant anti-HIV-1 activity with an IC_50_, a half-maximal inhibitory concentration, of 1.4 μM when tested on HIV-infected TZM-bl cells (also called JC.53bl-13) [35]. In addition, aspernigrin C from the same fungus also demonstrated similar action with an IC_50_ of 4.7 μM [35].

An anti-HIV bioassay conducted in 293T cells, also refered as a highly transfectable derivative of human embryonic kidney 293 cells, revealed that eutypellazine E, extracted from a fungus found in the depths of the ocean named *Eutypella* sp., significantly inhibited HIV-1 proliferation [36]. Furthermore, unlike truncateol P, truncateol O, which is derived from the ascomycete *Truncatella angustata*, was found to inhibit the replication of both the H1N1 and HIV-1 viruses [37]. In addition, penicillixanthone A which is derived from the fungus *Aspergillus fumigatus* that is native to jellyfish, has been shown to possess significant anti-HIV-1 activity by inhibiting the infection of CXCR4-tropic HIV-1 NL4-3 and CCR5-tropic HIV-1 SF162 [38]. Additionally, the fungus *Chaetomium globosum* found in the depths of the ocean was able to produce 1,3-dihydro-4,5,6-trihydroxy-7-methylisobenzofuran, epicoccone B, and xylariol [39]. They showed highly effective anti-HIV activity in vitro at the concentration of 20 μg/mL, with 75.10, 88.4, and 70.20% suppression rates, respectively [39].

Endophytic fungus metabolites have been demonstrated to possess a vast array of bioactivities, including anti-HIV properties. Phomonaphthalenone A and bostrycoidin, both of which were derived from the endophytic fungus *Phomopsis* sp., showed moderate anti-HIV activity and low cytotoxicity, with IC_50_ values of 11.6 and 9.4 μg/mL, respectively [40]. In addition, altertoxin I, II and III derived from the endophytic fungus *Alternaria tenuissima* QUE1Se inhibited HIV-1 virus replication completely [41]. The epoxyperylene structure of these molecules is a promising scaffold for the development of potent and non-toxic anti-HIV therapies [41]. Alternariol 5-O-methyl ether, on the other hand, was identified as a molecule that inhibits HIV-1 pre-integration processes after screening a library of bioactive compounds from the endophytic fungus *Colletotrichum* sp. [42]. Ergokonin A and B isolated from the endophytic fungus *Trichoderma* sp. Xy24 had IC_50_ values of 1.9 μM, which indicated that it significantly suppressed the HIV-1 virus [43]. Recently, it was discovered that the endophytic fungus *Phomopsis* sp. CGMCC No. 5416 produces phomopsone B and C, and these two phomopsones have significant antiviral activity, with IC_50_ values of 7.6 and 0.5 μmol/L, respectively [44]. Furthermore, the phenol pericochlorosin B, isolated from the endophytic fungus *Periconia* sp. F-31, showed significant anti-HIV activity in 293T cells, with an IC_50_ value of 2.2 μM [45]. In 2017, Pang et al. discovered four compounds produced by the plant endophytic fungus *Aspergillus* sp. That belong to phenalenone derivatives [46]. These compounds include asperphenalenone A and D, cytochalasin Z_8_, and epicocconigrone A, which have anti-HIV activities in vitro with IC_50_ values of 4.5, 2.4, 9.2, and 6.6 μM, respectively [46]. The endophytic fungus was isolated from the *Kadsura longipedunculata* plant, also known as the Chinese Kadsura Vine, and used in traditional Chinese medicine [46]. Lamivudine and efavirenz, two control positives, demonstrated a greater activity level, with IC_50_ values of 0.1 and 0.0004 μM, respectively [46].

## 3. Anti-Hepatitis Virus

The hepatitis virus is one of the major burdens on the global health system. There are currently numerous types of hepatitis virus, with both known and unknown etiologies [47,48]. Hepatitis C virus (HCV) and hepatitis B virus (HBV) are the most prevalent infectious agents linked to chronic liver disease, including hepatocellular carcinoma and cirrhosis [49,50]. In healthcare facilities, the use of contaminated blood poses a risk; the infection can be transmitted through unsafe injection practices, the injection of drugs, the transfusion of unscreened blood, and sexual practices involving blood inflammation [51].

### 3.1. Anti-Hepatitis C Virus

Chronic HCV infection affects approximately 71 million people, and approximately 400,000 people have died due to the infection, with 3–4 million new infections occurring each year [52]. Antiviral medications have been shown to cure approximately 95% of people infected with hepatitis C. The mechanism of action varies, but it involves the inhibition of viral-derived proteins, such as non-structural protein (NS)5A [53], NS5B [54], and NS3/4A [55]. Several direct acting antiviral drugs are currently available to combat HCV, including NS3/4A inhibitors (paritaprevir, asunaprevir, simeprevir, telaprevir, grazoprevir, and boceprevir), NS5A inhibitors (ledipasvir, ombitasvir, elbasvir, daclatasvir, and velpatasvir), and NS5B inhibitors (dasabuvir and sofosbuvir) [56]. However, these therapeutic drugs have some side effects and are quite expensive.

As shown in Table 1, a number of natural products produced by microorganisms have the potential to be developed into anti-HBV medications. It is widely believed that fungi represent one of the most promising sources of bioactive compounds from which anti-HBV drugs could be developed. In 1977, Marchelli and her colleagues purified for the first time a didehydropeptide, which was given the name NeoB. It is an abbreviation for neoechinulin B, which was isolated from the fungus *Aspergillus amstelodami* [57]. Nakajima and his colleagues later demonstrated that NeoB inhibited the development of infectious HCV in Huh-7 cells [58]. By inhibiting the liver X receptors (LXRs), its molecule improved the efficacy of all known anti-HCV drugs and demonstrated a significant synergistic effect when combined with either an HCV NS5A inhibitor or interferon [58]. To achieve high yields, Nishiuchi and his colleagues also developed the synthetic antiviral agent NeoB and other derivatives [20].

Natural products made by fungi that thrive in unique marine environments have also been particularly useful in drug discovery. Marine fungi have been the source of the discovery of many novel bioactive natural compounds with anticancer, antifungal, cytotoxic, and antibacterial properties for the past decade [59,60,61]. *Penicillium raistrickii* IMB17-034, a marine-derived fungus, was cultured to isolate raistrickindole A and raistrickin. Both chemicals inhibited Huh7.5 human liver cells infected with HCV, with EC_50_ values of 5.7 and 7.0 μM, respectively [62]. Harzianoic acid A and B are sesquiterpene-based analogues discovered in the symbiotic relationship of the *Trichoderma harzianum* ascomycete fungus with sponges [63]. These purified compounds demonstrated high efficacy in lowering HCV RNA levels in Huh7.5 cells [63]. Furthermore, both compounds are proposed to block HCV entry into the host, with potential targets including the viral E1/E2 and host cell CD81 proteins [63]. In 2016, Nishikori and his colleagues discovered peniciherquamide C, produced by *Penicillium herquei* P14190 and isolated from seaweed collected in Toba, Mie, Japan, after being incubated at 37 °C for 1–2 weeks [64]. Its anti-HCV molecule has an IC_50_ of 5.1 μM [64]. Furthermore, the cyclo (L-Tyr-L-Pro) diketopiperazine isolated from the endophytic fungus *Aspergillus versicolor* isolated from the Red Sea black sponge *Spongia officinalis* significantly inhibited HCV replication by inhibiting the activity of the HCV NS3/4A protease with an IC_50_ value of 8.2 μg/mL [65]. Similarly, an ethyl acetate extract of the fungus *Penicillium chrysogenum* obtained from the red alga *Liagora viscida* also secretes antiviral metabolites that inhibit the HCV NS3/4A protease [66].

Endophytic fungi have also been identified as a significant source of secondary metabolites, due to their complex and dynamic interactions with host plants [67]. A growing body of evidence suggests that endophytic fungi metabolites play an essential role in plant immunity against herbivores and pathogen defense and establish symbiosis with the host plant [67,68,69,70]. These secondary metabolites are expected to be a novel source of natural antiviral compounds, due to their diverse biological activities and wide structural variety. The activities of 44 endophytic fungi isolated from the Red Sea sponge *Hyrtios erectus* were studied and screened [71]. HCV inhibition was observed in extracts of *Penicillium chrysogenum* MERVA42, *Diaporthe rudis* MERVA25, *Auxarthron alboluteum* MERVA32, *Fusarium oxysporum* MERVA39, *Trichoderma harzianum* MERVA44, *Aspergillus versicolor* MERVA29, *Lophiostoma* sp. MERVA36, and *Penicillium polonicum* MERVA43 [71]. In addition, the HCV protease inhibitory activity of fourty-eight endophytic fungal strains isolated and purified from ten Egyptian medicinal plants was investigated. *Alternaria alternata* PGL-3, *Cochlibolus lunatus* PML-17, *Nigrospora sphaerica* EPS-38, and *Emerecilla nidulans* RPL-21 extracts inhibited the most HCV NS3/4A protease [72].

### 3.2. Anti-Hepatitis B Virus

People who are infected with HBV, of which there are over 350 million worldwide, are responsible for up to 80% of cases of primary liver cancer [73]. This disease is the leading cause of death worldwide. HBV infection may be responsible for 3% of total mortality in countries where HBV carrier rates reach 10%, a higher level than the mortality rate associated with polio before the introduction of the polio vaccine [74]. The WHO recommends the use of oral treatments, including tenofovir or entecavir, as the most potent drugs to suppress HBV [75]. A number of natural products produced by microorganisms, as shown in Table 1, have the potential to be developed into anti-HBV medications.

As part of the effort to discover new bioactive metabolites with anti-HBV properties from microbes, Ai and colleagues isolated 7-dehydroxyl-zinniol from *Alternaria solani*, an endophytic fungal strain found in the roots of the perennial herb *Aconitum transsectum*, which was shown to have moderate antiviral efficacy against HBV in the HBV-transfected HepG2.2.15 cell line (IC_50_ value of 0.38 μM), as evidenced by a decrease in hepatitis B surface antigen (HBsAg) secretion [76]. Furthermore, Jin and his colleagues investigated the secondary metabolite of the acidophilic fungus *Penicillium* sp. (strain OUCMDZ-4736) isolated from the root sediment of the mangrove *Acanthus ilicifolius*, also known as the holy mangrove [77]. Three new anthraquinone derivatives were successfully isolated from the low-pH fermentation broth of the OUCMDZ-4736 strain [77]. However, only two of them demonstrated anti-HBV activity, including 1-hydroxyisorhodoptilometrin and methyl 6,8-dihydroxy-3-methyl-9-oxo-9H-xanthene-1-carboxylate, which significantly inhibited HepG2.2.15 human hepatoblastoma cells with IC_50_ of 4.63 and 11.35 μM, respectively [77]. Both could prevent HepG2.2.15 cells from secreting HBsAg and (hepatitis B early antigen) HBeAg [77]. Regarding anti-HBV activity, both outperformed the positive control, lamivudine (IC_50_: 68.94 μM) [77]. Other derivatives produced by the OUCMDZ-4736 strain, on the other hand, did not show anti-HBV activity [77]. Another fungus, *Talaromyces* sp., produces secondary metabolites with anti-hepatitis properties, such as vanitaracin A. It is a tricyclic polyketide isolated from *Talaromyces* sp. broth. Vanitaracin A has potent anti-HBV activity in HBV-susceptible HepG2-hNTCP-C4 cells, with an IC_50_ value of 10.5 μM [78]. Furthermore, this molecule inhibits HBV viral entry signaling pathways in human hepatocytes. All HBV genotypes (A-D) were recognized by vanitaracin A, including a drug-resistant HBV isolate. According to these findings, vanitaracin A could be used in antiviral treatments to prevent HBV recurrence [79].

Even though pathogenic microbes, such as fungi, can cause severe diseases in hosts, many of them produce bioactive chemicals that could be used to develop new drugs [80,81,82]. Dong and colleagues investigated the anti-HBV properties of crude destruxins (a combination of cyclodepsipeptidic molecules, including destruxin A, B, and E, isolated from *Metarhizium anisopliae* var. *dcjhyium*, an entomopathogenic fungus that has a symbiotic relationship with the termite *Odontoternes formosanus* [83]. In HepG2.2.15 cells, these crude destruxins inhibited HBV-DNA replication, as well as HBsAg and HBeAg production [83]. An in vivo trial using ducks infected with duck HBV and treated for 15 days with crude destruxins revealed that the treated group had significantly lower levels of duck serum DHBV-DNA than the control group [83]. Furthermore, a pure form of destruxin B from the plant pathogenic fungus *Alternaria brassicae* suppresses HBsAg gene expression in human hepatoma Hep3B cells. Destruxin B had no negative effects on cell viability, implying that it could be developed in the future as a specialized anti-HBV medication [84].

## 4. Anti-Herpes Simplex Virus

The herpes simplex virus (HSV) causes a viral infection known as herpes. It can be spread orally via saliva, sores, and the mouth’s surface, or sexually via genital secretion or the mucocutaneous surface [85]. Both infections are mostly asymptomatic and go unnoticed, but they do cause a painful blister or ulcer at the site of infection [86]. It could be either mild or severe. Scientists have recently discovered HSV type 1 (HSV-1) and 2 (HSV-2). HSV-1 is transmitted mainly via oral-to-oral contact, whereas HSV-2 is primarily transmitted via genital-to-genital contact [87]. Symptoms are more severe in immunocompromised people, with more frequent recurrence. Herpes can also cause several complications, including encephalitis [88] and keratitis [89].

In 2016, according to the WHO, 3.7 billion people under 50 were infected with herpes [90]. The prevalence in Africa is approximately 88%, while 45% in America. Globally, genital herpes infection was estimated to affect 122 million to 192 million people under 50 [90]. Acyclovir [91], penciclovir [92], and valacyclovir are some of the drugs used to treat herpes [93]. However, these drugs cannot cure the disease; instead, they can only help reduce the severity and frequency of symptoms. As listed in Table 1, natural products made by microorganisms have the potential to be utilized in the development of anti-HSV medications.

As far as we know, the oceans are a rich source of natural compounds with antiviral properties, due to their unique aquatic habitat and vast biodiversity. Over 150 new alkaloids, sesquiterpenoids, polyketides, and other chemicals have been isolated from marine fungi [94]. A fungus isolated from fish gills, *Epicoccum nigrum* HDN17-88, produced amphiepicoccins, a new class of epipolythiodioxopiperazines. Amphiepicoccin A, C, and F exhibited anti-HSV-2 activity when tested in Vero cells using the cytopathic effect inhibition assay [95]. In addition, an independent group investigated the potency and mechanism of antiviral action of aspergillipeptide D, which was isolated from a culture broth of the marine gorgonian-derived fungus *Aspergillus* sp. SCSIO 41501 [96]. Aspergillipeptide D inhibited HSV-1 intercellular spread in Vero cells by lowering both the gene and protein levels of the viral late protein gB [96]. Furthermore, its compound has been reported to have an IC_50_ of 9.5 μM [97]. Additionally, the deep-sea fungus *Aspergillus versicolor* SCSIO 41502 was chemically analyzed, and 28 bioactive phenolic compounds were isolated. Aspergilol H and I, as well as coccoquinone A, were among the newly discovered metabolites that demonstrated antiviral activity against HSV-1 in the Vero cell line [98].

According to a report from Sun and his colleagues, *Trichobotrys effuse* DFFSCS021, isolated from deep-sea sediment in the South China Sea, produced novel tetramic acid derivatives known as trichobotrysins A-F. In Vero cell lines, trichobotrysin A, B, and D were found to be antiviral against HSV-1 [99]. Another study discovered seventeen bioactive compounds in the marine-derived fungus *Aspergillus terreus* SCSGAF0162 [100]. This fungus produced 11a-dehydroxyisoterreulactone A, arisugacin A, isobutyrolactone II, and aspernolide A, all of which had antiviral activity against HSV-1 in Vero cell lines [100]. Rowley and his team found antiviral activity in halovir A-E peptides extracted from the saltwater fermentation of the marine-derived fungus *Scytalidium* sp. The halovirs, linear and lipophilic peptides, had solid inhibitory activity against HSV-1 and HSV-2, as evidenced by their ability to inactivate the virus particle in HSV-infected Vero cells directly [101]. Furthermore, balticolid, a novel 12-membered macrolide, was discovered in a culture broth extract of an Ascomycetous fungus discovered in the Baltic Sea on the Greifswalder Bodden. Balticolid has an antiviral effect in Vero cells infected with HSV-1, according to in vitro tests [102].

As previously stated, fungal endophytes show promise as antimicrobial compounds to help combat drug resistance, antibiotic inefficiency, and the limited discovery of novel antimicrobial compounds [103]. Endophytic fungi produce antimicrobial substances with antibacterial, antifungal, antiprotozoal, and antiviral properties that aid in disease prevention [104,105]. A recent study tested endophytic fungi isolated from Egyptian medicinal plants for antiviral activity. HSV-2 was inhibited by 40.7% by an extract of the endophytic *Pleospora tarda*, originally isolated from the *Ephedra aphylla* medicinal plant [106]. Alternariol and alternariol-(9)-methyl ether have been identified as bioactive substances with antiviral properties [106]. Furthermore, researchers discovered that oblongolide Z isolated from the endophytic fungus *Phomopsis* sp. BCC 9789 has good activity against HSV type 1, with an IC_50_ of 14 μM [107]. This bioactive, however, has a cytotoxic effect on several cell lines, including KB, BC, NCI-H187, and Vero, with IC_50_ values of 37, 26, 32, and 60 μM, respectively [107].

Extremophilic fungi and endophytes have been discovered to develop novel mechanisms to survive in hostile environments, resulting in the production of novel natural compounds with diverse biological activities [108]. Exopolysaccharides 1 (EPS-1) and 2 (EPS-2) were isolated from thermotolerant *Bacillus licheniformis* and *Geobacillus thermodenitrificans* strains native to hot springs on Vulcano Island, Italy [109,110]. By modulating cytokine expression levels, EPS-1 and EPS-2 have antiviral properties that inhibit HSV-2 replication in human peripheral blood mononuclear cells [109,110].

Furthermore, entomopathogenic fungi are commonly used in agriculture as biological pest control agents. Many bioactive secondary metabolites have been isolated from pathogenic fungi strains, including pyridovericin, oxalic acid, beauveriolides, bassianin, beauvericins, tenellin, and oosporein [111,112]. The anti-HSV-1 activity of the 6,8-dihydroxy-3-hydroxymethyl isocoumarin, extracted from the pathogenic fungus *Torrubiella tenuis* BCC 12732 living on an insect’s scale, was modest, with an IC_50_ of 50 μg/mL determined using the green fluorescent protein (GFP)-based method [113]. Cordyol C, a novel diphenyl ether isolated from the insect-killing fungus *Cordyceps* sp. BCC 1861, was purified. Cordyol C demonstrated significant anti-HSV-1 activity when tested using a colorimetric method, with an IC_50_ of 1.3 μg/mL [114].

Bacteria are also known as natural product producers, and some of them have anti-HSV properties [115,116]. The engineered bacteria *Streptomyces hygroscopicus* 17997, which has a gdmP mutation, produced 4,5-dihydro-thiazinogeldanamycin, a novel geldanamycin derivative with significant anti-HSV-1 viral activity in Vero cells [117]. Another study discovered that a LabyA1, abbreviated from carbacyclic lantibiotic labyrinthopeptin A1, isolated from the actinomycete *Actinomadura namibiensis* DSM 6313, protects human embryonic lung-fibroblast cells from HSV particles. In addition, in vitro study show that LabyA1 has synergistic activity with clinically approved antiretroviral drugs like tenofovir, acyclovir, saquinavir, raltegravir, and enfuvirtide [118]. Furthermore, cyanobacteria are also known as a rich source of metabolites with a wide range of biological functions. The bioactivity of crude extracts of cyanobacteria isolated from estuaries in northern and central Portugal has been investigated [119]. A crude aqueous extract of *Leptolyngbya* sp. cyanobacteria was found to have anti-HSV1 activity in green monkey kidney (GMK) cells, implying that estuarine cyanobacteria could be used as an alternative in the search for new HSV-1 treatments [119].

Moreover, it has been reported that terrestrial fungi can also produce anti-HSV. New depsides containing monogalactopyranose isolated from the *Acremonium* sp. BCC 14080 fungus showed anti-HSV-1 activity in Vero cells, with an IC_50_ value of 7.2 μM [120]. Mellisol and 1,8-dihydroxynaphthol 1-O-glucopyranoside, two structurally distinct polyketides, were produced by the terrestrial fungus *Xylaria mellisii* (BCC 1005). Both compounds inhibited HSV-1 replication in Vero cells, with IC_50_ values of 10.50 and 8.40 μg/mL, respectively [121]. In addition, exocellular polysaccharide extracts derived from the fungus *Paecilomyces lilacinuson* were tested for anti-HSV-1 activity in mice [122]. Mice were infected with HSV-1 intracranially and given EPS extract intraperitoneally for seven days [122]. HSV-1 replication in the mouse brain was inhibited by EPS extracts in a dose-dependent manner [122]. Furthermore, the extracts significantly reduced the expression of nuclear factor kappa B (NF-κB) and tumor necrosis factor (TNF) in HSV-1-infected mouse brain tissue [122].

## 5. Anti-Influenza

The flu is a contagious respiratory illness caused by influenza viruses that infiltrate the nose, throat, and lungs. It can cause mild to severe illness and even death. Symptoms include fever, cough, sore throat, headache, fatigue, vomiting, and diarrhea. Human influenza A causes seasonal flu and has become a worldwide epidemic flu disease. This virus is classified into several subtypes based on the proteins on the virus’s surface layer known as hemagglutinin (H) and neuraminidase (N) [123]. Scientists have recently discovered 18 hemagglutinin subtypes (H1 to H18) and 11 neuraminidase subtypes (N1 to N11) [124]. H1N1 and H3N2 are the most common subtypes of influenza A circulating in humans [125]. The vaccine against influenza A is commercially available and protects against influenza viruses. It has been determined that the antiviral medications umifenevir and arbidol are effective in treating influenza A. These nucleoside antiviral drugs are directed toward the hemagglutinin envelope glycoprotein as their primary target [7]. Oseltamivir, also known as Tamiflu, is an additional medication that inhibits the neuraminidase of the influenza virus [7].

Natural products produced by microorganisms that could be investigated and used to develop anti-influenza drugs are depicted in Table 1. Spirostaphylotrichin X is a novel spirocyclic lactam isolated from the marine fungus *Cochliobolus lunatus* SCSIO41401 [126]. Spirostaphylotrichin X, with an IC_50_ value of 1.2 to 5.5 μM, demonstrated vigorous inhibitory activity against various influenza virus strains [126]. According to the mechanism of action, spirostaphylotrichin X inhibits influenza A virus replication by interfering with the activity of the PB2 protein [126]. In addition, the hybrid polyketide known as cladosin C, which was isolated from the deep-sea fungus *Cladosporium sphaerospermum* 2005-01-E3, contains a novel linear 6-enamino-7(8)-en-10-ol moiety with anti-influenza activity [127]. Furthermore, a marine actinobacterium known as *Verrucosispora* sp. MS100137 was responsible for the production of abyssomicin Y [128]. It is an abyssomicin of type I, and it has an epoxide group attached to the 8th and 9th carbon atoms in the structure. On oatmeal agar, *Verrucosispora* sp. was isolated from the sediment collected in April 2010 from a depth of 2733 m below sea level in the South China Sea, at the coordinates 20 degrees 9.795 inches north and 118 degrees 18.124 degrees east [128]. Abyssomicin Y has an IC_50_ of 8 μg/mL for anti-influenza A activity in A549 cells; however, ribavirin, a positive control drug, has only an IC_50_ of > 16 μg/mL.

In addition to marine fungi, extremophiles, such as acidophilic fungi, are a significant source of bioactive compounds and a potentially useful source of new anti-influenza medications. Purpurquinone B and C, purpurester A, and TAN-931 were isolated from the ethyl acetate extract of an acid-tolerant fungus *Penicillium purpurogenum* JS03-21 [129]. These compounds showed significant antiviral activity against H1N1, with IC_50_ values of 61.3, 64.0, 85.3, and 58.6 μM, respectively [129]. The mangrove-associated fungus *Diaporthe* sp. (SCSIO 41011) synthesized pestalotiopsone B and F, as well as 3,8-dihydroxy-6-methyl-9-oxo-9H-xanthene-1-carboxylate and 5-chloroisorotiorin all demonstrated significant anti-IAV activity against three different influenza A virus subtypes, including A/Puerto Rico/8/34 H274Y (H1N1), A/FM-1/1/47 (H1N1), and A/Aichi/2/68 (H3N2), with IC_50_ values of 2.52-39.97μM [130]. In addition, the aciduric fungal strain known as *Penicillium camemberti* OUCMDZ-1492 was isolated from an acidic marine niche, mangrove soil and mud, all of which were located close to the roots of *Rhizophora apiculate* [131]. Three indole-diterpenoids that had been previously isolated, including (2S,4bR,6aS,12bS,12cS,14aS)-3-deoxo-4b-deoxypaxilline, (2S,4aR,4bR,6aS,12bS,12cS,14aS)-4a-demethylpaspaline-4a-carboxylic acid, (2S,3R,4R,4aS,4bR,6aS,12bS,12cS,14aS)-4a-demethylpaspaline-3,4,4a-triol, in addition to two recently isolated indole-diterpenoids, including (2R,4bS,6aS,12bS,12cR,14aS)-9,10-diisopentenylpaxilline and (6S,7R,10E,14E)-16-(1H-indol-3-yl)-2,6,10,14-tetramethylhexadeca-2,10,14-triene-6,7-diol; and emindole SB, 21-isopentenylpaxilline, paspaline, and paxilline, were isolated from its fermentation broth at pH 5.0. These compounds demonstrated significant activity against the H1N1 virus, with IC_50_ values of 28.3, 38.9, 32.2, 73.3, 34.1, 26.2 μM, respectively [131]. The findings show that 3-oxo, 4-b-hydroxy, and 9-isopentenyl substitutions improve hexacyclic indole-diterpenoids’ anti-H1N1 activity [131]. Furthermore, the mangrove-derived fungus *Cladosporium* sp. PJX-41 produced molecules with anti H1N1, with IC_50_ values ranging from 82 to 89 μM, including (14S)-oxoglyantrypine, norquinadoline A, and four known alkaloid derivatives including deoxynortryptoquivaline, deoxytryptoquivaline, tryptoquivaline, and quinadoline B [132].

## 6. Anti-Respiratory Syncytial Virus

Respiratory syncytial virus, also known as RSV, is a member of the family paramyxoviridae and is a leading viral pathogen associated with the lower respiratory tract. RSV infections typically occur in infants and children worldwide [133]. Because it is a viral respiratory infection, it is the second leading cause of death overall [134]. This virus has also been connected to respiratory illnesses affecting the elderly and people with compromised immune systems. Most people experience a flu-like illness with a mild course as their primary manifestation. In severe cases, it can contribute to the development of bronchiolitis, also known as inflammation of the small lung airways, and pneumonia in children [135]. Two medications that are effective in treating RSV are palivizumab [136], and an aerosol form of ribavirin [137]. However, its application is restricted because it is toxic, expensive, and has highly variable efficacy. From the marine fungus *Aspergillus* sp strain XS-2009, Chen and his research groups isolated two natural products, namely 22-O-(N-Me-l-valyl)-21-epi-aflaquinolone B and aflaquinolones D, both of which have excellent anti-RSV activity in vitro; their IC_50_ values are 0.042 and 6.6 μM, respectively [138].

## 7. Anti-SARS-CoV-2

As of May 2022, the SARS-CoV-2 virus has infected over 510 million of people and killed over 6 million [10]. It has also wreaked havoc on the global economy and healthcare system [139]. Common symptoms of SARS-CoV-2 infection include headaches, fevers, fatigue, dry cough, dyspnea, diarrhea, chest pain, and muscle aches [140,141]. Moreover, some people experience anosmia and dysgeusia [142], as well as hemorrhagic and ischemic strokes [143].

As the pandemic continues, the availability of numerous efficient and safe vaccines has provided some relief [144,145,146]. A long list of potential COVID-19 drug candidates, each with their own mechanism of action, has been proposed [7,147,148,149]. Nevertheless, the US Food and Drug Administration has only approved two antiviral drugs for SARS-CoV-2, including remdesivir, a protease inhibitor, and baricitinib, a Janus kinase inhibitor that inhibits immune system overstimulation [150]. Remdesivir has the potential to be used to treat COVID-19 in both adults and children. In contrast, baricitinib treats COVID-19 in hospitalized adults who require supplemental oxygen, non-invasive or invasive mechanical ventilation, or extracorporeal membrane oxygenation [150,151]. However, the WHO only recommends baricitinib as a COVID-19 treatment [151]. Sotrovimab, a monoclonal antibody drug, has also been conditionally approved by the WHO to treat mild to moderate COVID-19 in patients at risk of hospitalization [151]. In spite of these encouraging developments, the development of additional therapeutics, such as small molecules, is necessary for controlling virus transmission and treating patients. A therapeutic approach that has proven effective against human viruses, including SARS-CoV-2, is the use of candidate molecules in combination regimens.

Given the slow rate of new compound discovery and development, repurposing or repositioning natural products to develop antiviral drug-inspired natural products against SARS-CoV-2 infection is becoming a more appealing proposition due to the use of well-characterized low-risk molecules, which may result in lower overall development costs and shorter development timelines.

A recent study published in 2022 found that the antimicrobial natural product aurasperone, as listed in Table 1, isolated from *Aspergillus niger* in the Red Sea tunicate *Phallusia nigra*, was highly effective against SARS-CoV-2 in vitro, with an IC_50_ of 12.25 μM. The IC_50_ result was comparable to the IC_50_ of the positive control remdesivir, which was 10.11 μM [152]. The in silico analysis revealed that the molecule aurasperone A targets M^pro^ in SARS-CoV-2 [152]. Furthermore, neoechinulin A isolated from *Aspergillus fumigatus* MR2012 from the Red Sea exhibited an IC_50_ value of 0.47 μM against SARS-CoV-2, with a similar target to M^pro^ [153]. NeoB, an anti-HBV alkaloid isolated from *A. amstelodami*, also demonstrated anti-SARS-CoV-2 activity, inhibiting liver X receptors [20]. It has a cytotoxicity threshold (CC_50_) of greater than 70 μM and an IC_50_ of 32.9 μM [20]. Furthermore, aspulvinone D, M, and R produced by *Cladosporium* sp. (7951) have IC_50_ values of 10.3; 9.4; and 7.7 μM, respectively, for inhibiting SARS-CoV-2 M^pro^. Previously, the fungus was isolated from *Paris polyphylla* var. *yunnanensis*, a medicinal plant collected in Kunming, China [154].

In addition, virtual screening and docking studies in aspergilol H, arisugacin A, aspernolide A, altertoxin V, cytochalasin Z_8_, (14S)-oxoglyantrypine, norquinadoline A, deoxynortryptoquivaline, and quinadoline B displayed a relatively high affinity to PL^pro^, 3CL^pro^, RNA-dependent RNA polymerase (RdRp), nsp15, and spike protein with binding energy ranging from −6.5 to −10 kcal/mol. Moreover, similar studies showed that 11a-dehydroxyisoterreulactone A displayed a relatively high affinity with 3CL^pro^ of SARS-CoV-2, with a binding energy of -8.9 kcal/mol. Furthermore, isobutyrolactone and aspernolide A bind to M^pro^ of SARS-CoV-2 via a critical hydrogen bond interaction with Gly143 and Thr415, respectively [155]. RdRP of SARS-CoV-2 showed that alternariol and alternariol-(9)-methyl ether have binding energies of −7.6 and −8.5 kcal/mol, respectively [156]. A similar study of an anti-HSV cyclic peptide, aspergillipeptide D, revealed inhibitory activity against SARS-CoV-2, with M^pro^ as a target [157]. It then inspired the synthesis and development of five oxazole-based macrocycles with inhibitory activity against SARS-CoV-2 (NRC-03-nhCoV) in Vero-E6 cells, with an IC_50_ of 18.3–63.3 μM [157].

## 8. Viruses in Their Biological Make-Up and Possible Life Cycle Target

**Figure 1:**
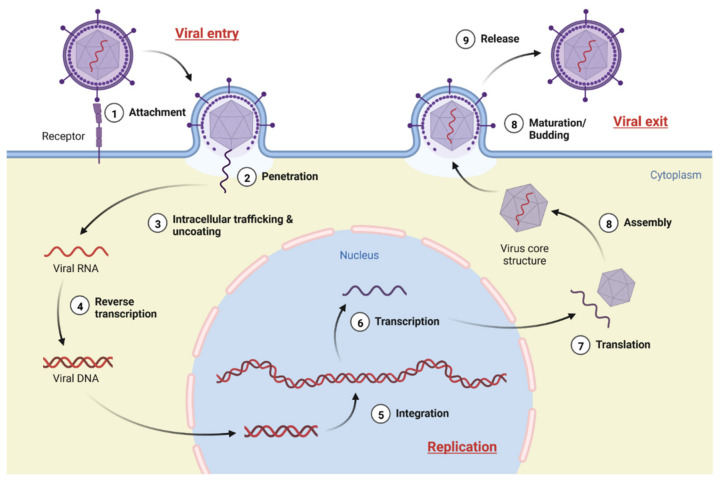
General illustration of virus life cycle (created with BioRender.com, accessed on 29 May 2022).

Viruses are intracellular parasites that inhabit the cells of their host [158]. In order for viruses to produce their progeny, they first must penetrate the cells they are targeting and then seize control cellular machinery of the host. The process is only possible if viruses have successfully entered their target cells. “Life cycle” refers to the process a virus undergoes in order to replicate within a host cell. In general, the life cycle of a virus is comprised of three distinct phases, including entry, genome replication, and exit, as shown in Figure 1.

### 8.1. Viral Entry

#### 8.1.1. Mechanism

Entry of a virus is the initial phase of an infection. It describes the interaction between a membrane protein of the virus particle and a viral receptor. The four steps of viral entry are attachment, penetration, intracellular trafficking, and uncoating [159]. Attachment refers to the initial contact of virus particles with the host cells. This process occurs at the plasma membrane of the host cell and requires attachment factors and viral receptors. The cell surface attachment factor is accountable for recruiting and retaining virus particles; facilitating the interaction between the viral particle and the host receptor. In general, viruses subvert the physiological functions of cellular proteins and use them as entry receptors in the host [160]. As shown in Table 2, each subtype of virus has a unique host receptor. For example, the life cycle of HCV begins with virus particle attachment to the host via interaction of the E1/E2 heterodimer membrane protein with cluster of differentiation 81 (CD81) and scavenger receptor class B type 1 (SRB1) attachment factor [161].

In HIV, the virus particle recognizes CD4, a member of the immunoglobulin superfamily, in conjunction with chemokine coreceptors, such as CCR5 or CXCR4, and an attachment factor called dendritic cell-specific intercellular adhesion molecule-3-grabbing non-integrin (DC-SIGN) [162,163]. In the case of naked viruses, such as poliovirus and norovirus, capsid proteins may bind to the receptor directly [164]. In addition, heparan sulfate proteoglycans (HSPG) serve as the attachment factor for a variety of viruses, such as HBV, RSV, and HSV [165], as shown in Table 2, illustrating the broader specificity of attachment factors. Once successful attachment has occurred, the virus particle can infect the host. In the case of HCV, virus particles are capable of penetrating the tight junction of hepatocytes [166]. In the case of HIV, virus particles penetrate T helper lymphocytes [163].

Once the virus has penetrated the host cell, the signalling pathway promotes the formation of endocytic vesicles, also known as receptor-mediated endocytosis [167]. For instance, the virus particle serves as the endocytosis ligand [167]. Most viruses, except retrovirus, depend on endocytic uptakes [168,169]. Additionally, endocytosis enables viruses to avoid leaving the viral envelope glycoprotein on the plasma membrane, which delays host immune detection [170]. The acidic pH of endosomes triggers the fusion of viral envelope with endosomal membrane for enveloped viruses, also called membrane fusion [171]. In the case of unenveloped naked viruses, one of the capsid proteins induces endosome lysis, which is also called membrane lysis [172]. For instance, in the case of HCV, CD81 interacts with claudin 1 (CLDN1) to initiate endocytosis in response to clathrin stimulation [173]. This process is referred to as clathrin-mediated endocytosis [173]. Thus, positive-strand RNA is released from the endosome into the cytosol [173]. This mechanism exists in influenza viruses as well [174].

After successfully penetrating the cell, the virus particles must travel to a suitable location to replicate their genome. This process is known as intracellular trafficking [175]. Some viruses replicate in the cytoplasm, i.e., HCV [176], while others do so through a nuclear pore. Replication through the nuclear pore, i.e., HSV and influenza virus, necessitates a variety of distinct strategies, the majority of which are determined by the size of the genome [177]. HSV nucleocapsids are mildly disassembled to permit entry of the DNA genome into the nucleus [178]. Nucleocapsids must, therefore, be directed to the replication site. This mechanism can occur via microtubule-mediated transport [179]. During the movement of viruses from the cell periphery to the perinuclear space, the viral genome must be uncoated by viral enzymes or host enzymes, releasing the viral genomic nucleic acid. Uncoating is dependent on endocytic and cytoplasmic pathways and trafficking [180].

#### 8.1.2. Possible Target of Viral Entry

**Figure 2:**
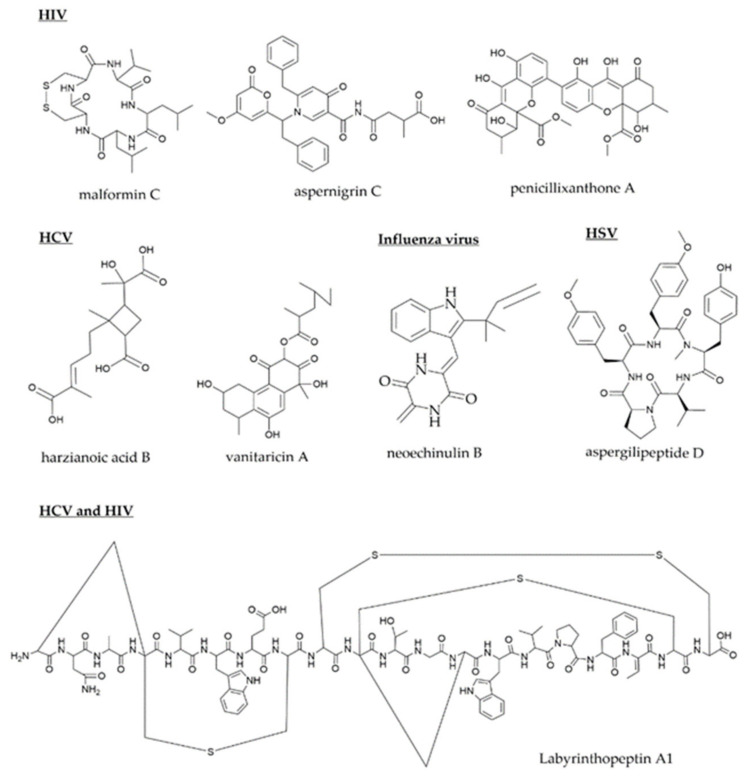
Possible target of viral entry in viruses using microbial natural products.

As described in Section 2, Section 3, Section 4, Section 5, Section 6 and Section 7, multiple molecules or natural products (NPs) were discovered and obtained from microbes, followed by an IC_50_/EC_50_ analysis. However, only a few molecules have been studied in terms of mode of action [181]. Drugs can be developed from molecules by understanding the mechanism of action [182]. Malformin C, aspernigrin C, and penicillixanthone A are three HIV life cycle inhibitors that target viral entry, as shown in Figure 2 [35,38]. Malformin C and Aspernigrin C could inhibit CCR5 during the HIV-1 entry [35,183]. In addition, penicillixanthone A also inhibits the HIV entry by inhibiting CCR5 and CXCR4 [182,184]. Dual co-receptor antagonists, such as penicillixanthone, are beneficial for drug development because HIV resistance can be acquired by switching from CCR5 to CXCR4, and this molecule inhibits both co-receptors. Furthermore, two molecules, harzianoic A and B, have been shown to inhibit HBV entry by targeting CD81 proteins [63]. Additionally, microbe-produced vanitaricin A can interact directly with the sodium taurocholate cotransporting polypeptide (NTCP) in HBV, impairing bile acid transport [78,79]. In addition, neoechinulin B bound to the influenza (H1N1) envelope haemagglutinin and disrupted its interaction with the host sialic acid receptor, preventing the influenza virus from attaching to host cells [20,185]. Labyrinthopeptin A prevents both HIV and HSV entry. In HSV and HIV, it targets glycoprotein receptors and CD4 cells, respectively. In contrast, the molecule has no effect on CXCR4 or CCR5 [78]. Unlike all of these molecules, aspergillipeptide D inhibit the synthesis of gB protein, which will reduce the intracellular spread of HSV-1. In contrast to these other molecules, aspergillipeptide D inhibits the synthesis of gB protein, thereby reducing the intracellular spread of HSV-1. The glycoproteins gD, gH/gL, and gB are primarily responsible for direct membrane fusion and spread [96].

### 8.2. Genome Replication

#### 8.2.1. Mechanism

As previously stated, some viruses replicate in the cytoplasm, while others do so via a nuclear pore. In the case of HSV, the positive-strand RNA is released into the cytosol upon its release [186]. When ribosomal subunit binds to an RNA particle in the rough endoplasmic reticulum (ER), polyprotein translation is then initiated [187]. Subsequently, the ribosome–RNA complex attaches to the ER membrane, completing the translation of HCV polyprotein [187]. A single polyprotein of approximately 3000 amino acids is generated by the translation process [188]. Thus, proteolytic processing of viral proteins occurs within rough ER. It cleaves the core, E1, E2, and P7 proteins with its protease [189]. Following this, the remaining proteins are cleaved. The end result is ten mature HCV proteins, including four structural proteins (core, E1, E2, and P7) and six additional non-structural proteins (NS2, NS3, NS4A, NS4B, NS5A, and NS5B) [189]. As shown in Table 2, SARS-CoV-2 contains 16 non-structural proteins (NSP1 to NSP16). Two cysteine proteases, M^pro^ (or 3CL^pro^) and papain-like protease (PL^pro^), are responsible for this extensive proteolytic processing [7]. The release of NSP1 to NSP3 from the N-terminus of polyproteins is the responsibility of PL^pro^ [190]. Starting with the autolytic cleavage of this enzyme (NSP5) from the polyproteins pp1a and pp1b, M^pro^ digests the polyprotein at the remaining 11 conserved cleavage sites (NSP4 to NSP16) [190]. In HCV, replication occurs in the membrane web, and NS5B RdRp catalyzes the synthesis of a negative-sense RNA intermediate (template) that is used to generate multiple copies of positive-sense progeny HCV RNA [191]. This newly synthesized HCV RNA is either incorporated into nucleocapsid particles or utilized for RNA translation and replication [191]. Multiple HCV non-structural proteins facilitate the replication of RNA [192].

#### 8.2.2. Possible Target

**Figure 3:**
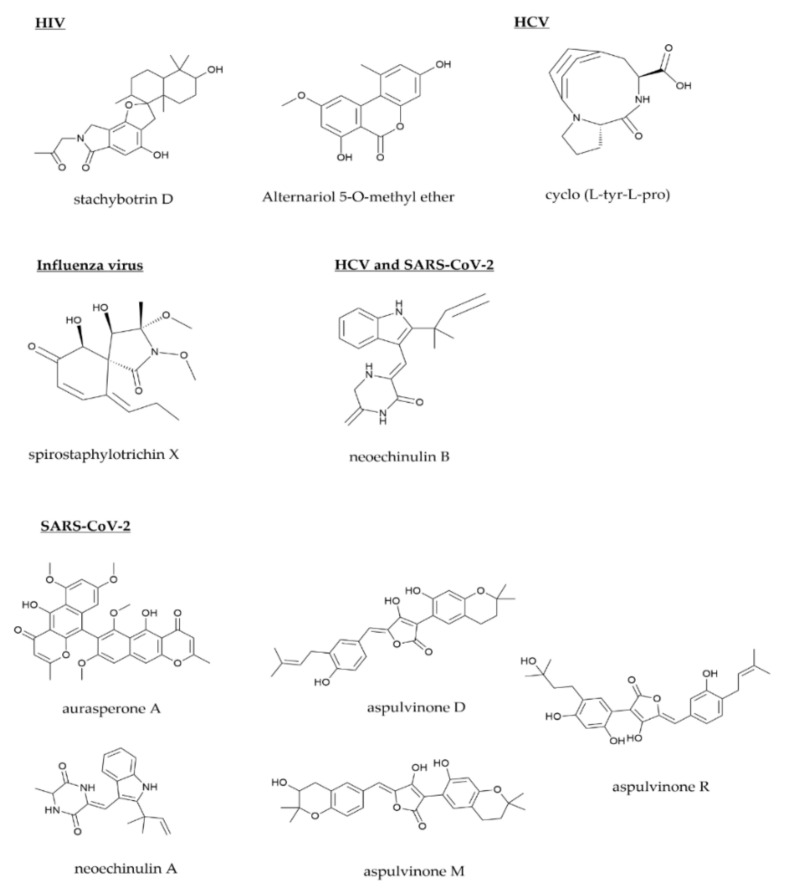
Possible target of replication in viruses using microbial natural products.

As depicted in the Figure 3, two molecules serve as inhibitors of HIV replication, stachybotrin and alternariol 5-O-methyl ether. Stachybotrin D inhibited HIV-1 replication without cytotoxicity, as shown by anti-HIV testing of the compounds. It has been reported that it inhibits HIV-1 NRT (non-nucleoside reverse transcriptase) [32]. Stachybotrin D is a promising molecule because its structure differs substantially from that of the currently available NRT drugs, such as etravirine, rilpivirine, doravirine, nevirapine, and efavirenz. It is devoid of cyclopropyl and alkynyl groups and contains only non-aromatic nitrogen [32]. Additionally, alternariol 5-O methyl ether inhibits integration by preventing nuclear import of the pre-integration complex, which is essential for HIV-1 replication [42]. The cyclo (L-tyr-L-pro) produced by *A. versicolor* could inhibit NS3/4A that is essential for HCV replication [65] and suppress the host viral immune system [193]. The polymerase basic 2 (PB2) subunit is involved in the initiation of transcription and replication of the influenza virus genome. By interfering with the activity of PB2 protein, spirostaphylotrichin 1 can inhibit the replication of influenza A virus [126]. In addition to acting as an entry inhibitor, it has been confirmed that neoechinulin B inhibits HCV replication by inhibiting liver X receptors (LXRs) [58]. It is also capable of inhibiting the transcriptional activity of LXRs in SARS-CoV-2. This disrupts the formation of double-membrane vesicles, the sites of viral RNA replication. This decreases viral replication in infected cells [20].

M^pro^ plays an essential role in the replication and transcription of SARS-CoV-2. There are a number of microbe-produced natural products with the ability to inhibit M^pro^, including aurasperone A [152]; neoechinulin A [153]; as well as aspulvinone D, M, and R [154].

### 8.3. Viral Exit

Three steps comprise the exit process, capsid assembly, release, and maturation.

#### 8.3.1. Assembly and Maturation

Two processes comprise capsid assembly, capsid assembly in the ER and genome packaging in the Golgi complex [194]. Depending on the virus, these two processes may occur sequentially or simultaneously [195]. Additionally, maturation is the final stage of virus particle assembly [196]. In the case of HIV, the cleavage of the Gag polyprotein by the viral aspartate protease is accompanied by a significant morphological change, such as the condensation of the capsid structure [197]. Importantly, this maturation process imparts infectious potential to the particle.

#### 8.3.2. Release

The release of virus particles from naked viruses results from the lysis of infected cells. Since the cell membrane that served as a trap for the virus particles has been destroyed, no specific exit mechanism is required [198]. Prior to the release of enveloped viruses, the capsids go through an envelopment process in which they become surrounded by a lipid bilayer [199]. Exocytosis, which takes place at the conclusion of the exit step, permits the majority of enveloped viruses to escape from cells. This procedure, which is initiated by late domains, is also known as budding [200]. Therefore, no specific molecule produced by microbes with an exit-related mode of action has been identified.

## 9. Conclusions and Outlook

This literature review suggests that microbes produce natural products with diverse antiviral activities, including SARS-CoV-2, and various mechanisms of action. Based on the analysis, fungi, particularly Ascomycota, are a rich source of antiviral molecules that are distinct from those found in bacteria and microalgae. A possible explanation is that fungi, including Ascomycota, have larger genomes with diverse biosynthetic gene clusters (BGCs) [201]. Moreover, according to the analysis, extreme habitats, such as oceans and mangrove ecosystems, contribute to forming natural product patterns, which are recognized as a promising source of structurally novel and diverse antiviral compounds. Endophytes and the group of pathogenic fungi also contribute to producing antiviral compounds. Endophytic fungi, which interact with host plants and cross-communicate with other endophytic microbes colonizing the same plant, probably induce chemical signaling and chemical defense against different microorganisms, including viruses. Some can also protect host plants from pathogens by imitating plant defense natural products. In addition, pathogens, such as plant pathogens or entomopathogens, produce chemical attacks or defenses with unprecedented molecules. Specific molecules may be repurposed for use in additional antiviral activities by first undergoing virtual screening and docking studies, followed by in vitro validation of their efficacy.

Recent research has shown that the newly created synthetic compound MM3122 is effective against a variety of viruses [202]. Not only is it effective against SARS-CoV-2, but also against infections caused by the majority or all coronaviruses and influenza viruses [202]. Moreover, in vitro research has revealed that certain microbial natural products can inhibit multiple viruses, including labyrinthopeptin A1, which can prevent viral entry in HSV and HIV [118]. In addition, it has been reported that truncateol O could inhibit HIV and influenza viruses via an undefined mechanism [37]. Furthermore, neoechinulin B inhibits the replication of the HCV, SARS-CoV-2, and influenza viruses [58,185]. A new method for synthesizing neoechinulin B and its derivatives has also been developed [20]. Thus, it is possible that these natural products could be developed in a similar fashion to MM3122. 

Natural products produced by microbes, as described in the previous section, target viral entry in the life cycle more frequently than viral replication. Additionally, to the best of our knowledge, no target inhibition for viral exit has been described. Furthermore, the majority of natural products, as is the case with the majority of antiviral drugs, target a similar pathway. In vitro tests show that natural products are as efficacious as commercially available drugs. The IC_50_ values for aurasperone A (12.25 μM) were comparable to those for remdesivir (10.11 μM) [152]. Both have a mechanism of action that inhibits M^pro^, which is essential for the replication and transcription of SARS-CoV-2 [152]. In addition, methyl 6,8-dihydroxy-3-methyl-9-oxo-9H-xanthene-1-carboxylate outperformed the positive control, lamivudine (IC_50_: 68.94 μM), with an IC_50_ of 11.35 μM [130]. Another study demonstrated that labyrinthopeptin A1 has a synergistic effect with clinically approved antiretroviral medications, such as raltegravir, enfuvirtide, acyclovir, saquinavir, and tenofovir [118]. Based on the analysis of the gathered data, it is possible for microbes to produce natural products that target the life cycle of multiple viruses; therefore, advanced laboratory research must be conducted with care. Moreover, there are still few studies pertaining to virus exit. In addition to entry and replication inhibitors, this may be a good target that researchers have overlooked. According to in vitro data, the inhibitory activity of the natural product is comparable to that of commercially available drugs. In addition, it can exhibit synergistic mechanisms with other molecules. Thus, microbial natural products hold great promise for the development of antiviral drugs. It would be intriguing to evaluate the molecule in vivo and through additional advancement research in the near future.

## Figures and Tables

**Table 1:** Natural product produce by microbes and its target.

Compound Name [Ref.]	Compound Type	Microbial Strain	Strain Origin/Host	Viral Target	IC_50_/EC_50_/ED_50_	Target Inhibition
alachalasin A [25]	alkaloid	*Podospora vesticola* XJ03-56-1	glacier	HIV-1	EC_50_ = 8.01 μM	ND
pestalofone A [28]	terpenoid	*Pestalotiopsis fici* W106-1	plant endophyte	HIV-1	EC_50_ = 90.4 μM	ND
pestalofone B [28]	terpenoid	*P. fici* W106-1	plant endophyte	HIV-1	EC_50_ = 64.0 μM	ND
pestalofone E [28]	terpenoid	*P. fici* W106-2	plant endophyte	HIV-1	EC_50_ = 93.7 μM	ND
pestaloficiol G [28]	terpenoid	*P. fici* W106-3	plant endophyte	HIV-1	EC_50_ = 89.2 μM	ND
pestaloficiol H [28]	terpenoid	*P. fici* W106-4	plant endophyte	HIV-1	EC_50_ = 89.2 μM	ND
pestaloficiol J [28]	terpenoid	*P. fici* W106-5	plant endophyte	HIV-1	EC_50_ = 8 μM	ND
pestaloficiol K [28]	terpenoid	*P. fici* W106-6	plant endophyte	HIV-1	EC_50_ = 78.2 μM	ND
epicoccin G [95]	alkaloid	*Epicoccum nigrum* XZC04-CS-302	*Cordyceps sinensis* fungus	HIV-1	EC_50_ = 13.5 μM	ND
epicoccin H [95]	alkaloid	*E. nigrum* XZC04-CS-302	*C. sinensis*	HIV-1	EC_50_ = 42.2 μM	ND
diphenylalazine A [95]	peptide	*E. nigrum* XZC04-CS-302	*C. sinensis*	HIV-1	EC_50_ = 27.9 μM	ND
bacillamide B [30]	peptide	*Tricladium sp.* No. 2520	soil in which *C. sinensis* grow	HIV-1	EC_50_ = 24.8 μM	ND
armochaetoglobin K [31]	alkaloid	*Chaetomium globosum* TW 1-1	*Armadillidium vulgare* insect	HIV-1	EC_50_ = 1.23 μM	ND
armochaetoglobin L [31]	alkaloid	*C. globosum* TW 1-1	*A. vulgare* insect	HIV-1	EC_50_ = 0.48 μM	ND
armochaetoglobin M [31]	alkaloid	*C. globosum* TW 1-1	*A. vulgare* insect	HIV-1	EC_50_ = 0.55μM	ND
armochaetoglobin N [31]	alkaloid	*C. globosum* TW 1-1	*A. vulgare* insect	HIV-1	EC_50_ = 0.25 μM	ND
armochaetoglobin O [31]	alkaloid	*C. globosum* TW 1-1	*A. vulgare* insect	HIV-1	EC_50_ = 0.61 μM	ND
armochaetoglobin P [31]	alkaloid	*C. globosum* TW 1-1	*A. vulgare* insect	HIV-1	EC_50_ = 0.68 μM	ND
armochaetoglobin Q [31]	alkaloid	*C. globosum* TW 1-1	*A. vulgare* insect	HIV-1	EC_50_ = 0.31 μM	ND
armochaetoglobin R [31]	alkaloid	*C. globosum* TW 1-1	*A. vulgare* insect	HIV-1	EC_50_ = 0.34 μM	ND
stachybotrin D [32]	terpenoid	*Stachybotrys chartarum* MXH-X73	*Xestospongia testudinaris* sponge	HIV-1	EC_50_ = 8.4 μM	replication
stachybotrysam A [33]	alkaloid	*S. chartarum* CGMCC 3.5365.	ND	HIV-1	EC_50_ = 9.3 μM	ND
stachybotrysam B [33]	alkaloid	*S. chartarum* CGMCC 3.5365.	ND	HIV-1	EC_50_ = 1.0 μM	ND
stachybotrysam C [33]	alkaloid	*S. chartarum* CGMCC 3.5365.	ND	HIV-1	EC_50_ = 9.6 μM	ND
chartarutine B [34]	alkaloid	*S. chartarum* WGC-25C-6	*Niphates* sp. sponge	HIV-1	IC_50_ = 4.90 μM	ND
chartarutine G [34]	alkaloid	*S. chartarum* WGC-25C-6	*Niphates* sp. sponge	HIV-1	IC_50_ = 5.57 μM	ND
chartarutine H [34]	alkaloid	*S. chartarum* WGC-25C-6	*Niphates* sp. sponge	HIV-1	IC_50_ = 5.58 μM	ND
malformin C [35]	peptide	*Aspergillus niger* SCSIO Jcsw6F30	marine	HIV-1	IC_50_ = 1.4 μM	entry
aspernigrin C [181]	alkaloid	*A. niger* SCSIO Jcsw6F30	marine	HIV-1	IC_50_ = 4.7 μM	entry
eutypellazine E [36]	alkaloid	*Eutypella* sp. MCCC 3A00281	deep sea sediment	HIV-1	IC_50_ = 3.2 μM	ND
truncateol O [37]	terpenoid	*Truncatella angustata* XSB-01-43	*Amphimedon* sp. sponge	HIV-1 and H1N1	IC_50_ = 39.0 μM (HIV) and 30.4 μM (H1N1)	ND
truncateol P [37]	terpenoid	*T. angustata* XSB-01-43	*Amphimedon* sp. sponge	HIV-1	IC_50_ = 16.1 μM	ND
penicillixanthone A [38]	polyketide	*Aspergillus fumigatus*	jellyfish	HIV-1	IC_50_ = 0.26 μM	entry
DTM [39]	polyketide	*C. globosum*	deep sea sediment	HIV-1	75.1% at 20 μg/mL	ND
epicoccone B [39]	polyketide	*C. globosum*	deep sea sediment	HIV-1	88.4% at 20 μg/mL	ND
xylariol [39]	polyketide	*C. globosum*	deep sea sediment	HIV-1	70.2% at 20 μg/mL	ND
phomonaphthalenone A [40]	polyketide	*Phomopsis sp.* HCCB04730	*Stephania japonica*-plant endophyte	HIV-1	IC_50_: 11.6 μg/mL	ND
bostrycoidin [40]	polyketide	*Phomopsis sp.* HCCB04730	*S. japonica* plant endophyte	HIV-1	IC_50_: 9.4 μg/mL	ND
altertoxin I [41]	phenalene	*Alternaria tenuissima* QUE1Se	*Quercus emoryi* plant endophyte	HIV-1	IC_50_: 1.42 μM	ND
altertoxin II [41]	phenalene	*A. tenuissima* QUE1Se	*Q. emoryi* plant endophyte	HIV-1	IC_50_: 0.21 μM	ND
altertoxin III [41]	phenalene	*A. tenuissima* QUE1Se	*Q. emoryi* plant endophyte	HIV-1	IC_50_: 0.29 μM	ND
alternariol 5-O-methyl ether [42]	phenolic	*Colletotrichum* sp	plant endophyte	HIV-1	EC_50_: 30.9 μM	replication
ergokonin A [43]	terpenoid	*Trichoderma* sp. Xy24	*Xylocarpus granatum* plant endophyte	HIV-1	IC_50_: 22.3 μM	ND
ergokonin B [43]	terpenoid	*Trichoderma* sp. Xy24	*X. granatum* plant endophyte	HIV-1	IC_50_: 1.9 μM	ND
sorrentanone [43]	terpenoid	*Trichoderma* sp. Xy24	*X. granatum* plant endophyte	HIV-1	IC_50_: 4.7 μM	ND
cerevisterol [43]	terpenoid	*Trichoderma* sp. Xy24	*X. granatum* plant endophyte	HIV-1	IC_50_: 9.3 μM	ND
phomopsone B [44]	alkaloid	*Phomopsis* sp. CGMCC 5416	*Achyranthes bidentata* plant endophyte	HIV-1	IC_50_: 7.6 μmol/L	ND
phomopsone C [44]	alkaloid	*Phomopsis* sp. CGMCC 5416	*A. bidentata* plant endophyte	HIV-1	IC_50_: 0.5 μmol/L	ND
pericochlorosin B [45]	polyketide	*Periconia* sp. F-31	plant endophyte	HIV-1	IC_50_: 2.2 μM	ND
asperphenalenone A [46]	alkaloid	*Aspergillus* sp.	*Kadsura longipedunculata* plant endophyte	HIV-1	IC_50_: 4.5 μM	ND
asperphenalenone D [46]	alkaloid	*Aspergillus* sp.	*K. longipedunculata* plant endophyte	HIV-1	IC_50_: 2.4 μM	ND
cytochalasin Z_8_ [46]	alkaloid	*Aspergillus* sp.	*K. longipedunculata* plant endophyte	HIV-1	IC_50_: 9.2 μM	ND
epicocconigrone A [46]	alkaloid	*Aspergillus* sp.	*K. longipedunculata* plant endophyte	HIV-1	IC_50_: 6.6 μM	ND
neoechinulin B/NeoB [57,153,185]	alkaloid	*Aspergillus amstelodami*	ND	HCV and SARS-CoV-2	IC_50_: 5.5 μM (HCV) and 32.9 μM (SARS-CoV-2)	replication
		*Eurotium rubrum F33*	marine sediment	H1N1	IC50; 7 μM	entry
raistrickindole A [62]	alkaloid	*Penicillium raistrickii* IMB17-034	mangrove sediment	HCV	EC_50_: 5.7 μM	ND
raistrickin [62]	alkaloid	*P. raistrickii* IMB17-035	mangrove sediment	HCV	EC_50_: 7.0 μM	ND
sclerotigenin [62]	alkaloid	*P. raistrickii* IMB17-036	mangrove sediment	HCV	EC_50_: 5.8 μM	ND
harzianoic acid A [43]	terpenoid	*Trichoderma harzianum* LZDX-32-08	*Xestospongia testudinaria* sponge	HCV	IC_50_: 5.5 μM	entry
harzianoic acid B [43]	terpenoid	*T. harzianum* LZDX-32-08	*X. testudinaria* sponge	HCV	IC_50_: 42.9 μM	entry
peniciherquamide C [64]	peptide	*Penicillium herquei* P14190	seaweed	HCV	IC_50:_ 5.1 μM	ND
cyclo (L-Tyr-L-Pro) [65]	peptide	*Aspergillus versicolor*	*Spongia officinalis* sponge	HCV	IC_50_: 8.2 μg/mL	replication
7-dehydroxyl-zinniol [76]	alkaloid	*Alternia solani*	*Aconitum transsectum* plant endophyte	HBV	IC_50_: 0.38 mM	ND
THA [77]	polyketide	*Penicillium* sp. OUCMDZ-4736	mangrove sediment	HBV	IC_50_: 4.63 μM	ND
MDMX [77]	polyketide	*Penicillium* sp. OUCMDZ-4736	*mangrove* sediment	HBV	IC_50_: 11.35 μM	ND
vanitaracin A [78]	polyketide	*Talaromyces* sp.	sand	HBV	IC_50_: 10.58 μM	entry
destruxin A [83]	peptide	*Metarhizium anisopliae* var. *dcjhyium*	*Odontoternes formosanus* termite	HBV	IC_50_: 1.2 μg/mL (mix A+B+E)	ND
destruxin B [83]	peptide	*M. anisopliae* var. *dcjhyium;*	*O. formosanus* termite	HBV	IC_50_: 1.2 μg/mL (mix A+B+E)	ND
destruxin E [83]	peptide	*M. anisopliae* var. *dcjhyium*	*O. formosanus* termite	HBV	IC_50_: 1.2 μg/mL (mix A+B+E)	ND
amphiepicoccin A [95]	alkaloid	*Epicoccum nigrum* HDN17-88	*Amphilophus* sp. fish gill	HSV-2	IC_50_: 70 μM	ND
amphiepicoccin C [95]	alkaloid	*E. nigrum* HDN17-88	*Amphilophus* sp. fish gill	HSV-2	IC_50_: 64 μM	ND
amphiepicoccin F [95]	alkaloid	*E. nigrum* HDN17-88	*Amphilophus* sp. fish gill	HSV-2	IC_50:_ 29 μM	ND
aspergillipeptide D [96]	peptide	*Aspergillus* sp. SCSIO 41501	gorgonian coral	HSV-1	IC_50_: 7.93 μM	entry
aspergilol H [98]	polyketide	*Aspergillus versicolor* SCSIO 41501	deep sea sediment	HSV-1	EC_50_ = 4.68 μM	ND
aspergilol I [98]	polyketide	*A. versicolor* SCSIO 41503	deep sea sediment	HSV-1	IC_50_ = 6.25 μM	ND
coccoquinone A [98]	polyketide	*A. versicolor* SCSIO 41504	deep sea sediment	HSV-1	IC_50_ = 3.12 μM	ND
trichobotrysin A [99]	alkaloid	*Trichobotrys effuse* DFFSCS021	deep sea sediment	HSV-1	IC_50_ = 3.08 μM	ND
trichobotrysin B [99]	alkaloid	*Trichobotrys effuse* DFFSCS021	deep sea sediment	HSV-1	IC_50_ = 9.37 μM	ND
trichobotrysin D [99]	alkaloid	*Trichobotrys effuse* DFFSCS021	deep sea sediment	HSV-1	IC_50_ = 3.12 μM	ND
11a-dehydroxyisoterreulactone A [100]	terpenoid	*Aspergillus terreus* SCSGAF0162	gorgonian corals *Echinogorgia aurantiaca*	HSV-1	IC_50_ = 16.4 μg/mL	ND
arisugacin A [100]	terpenoid	*Aspergillus terreus* SCSGAF0162	gorgonian corals *E. aurantiaca*	HSV-1	IC_50_ = 6.34 μg/mL	ND
isobutyrolactone II [100]	terpenoid	*Aspergillus terreus* SCSGAF0162	gorgonian corals *E. aurantiaca*	HSV-1	IC_50_ = 21.8 μg/mL	ND
aspernolide A [100]	terpenoid	*Aspergillus terreus* SCSGAF0162	gorgonian corals *E. aurantiaca*	HSV-1	IC_50_ = 28.9 μg/mL	ND
halovir A [101]	peptide	*Scytalidium* sp.	NI	HSV-1 and HSV-2	ED_50_ = 1.1 μM (HSV-1) and 0.28 (HSV-2)	ND
halovir B [101]	peptide	*Scytalidium* sp.	NI	HSV-1	ED_50_ = 3.5 μM	ND
halovir C [101]	peptide	*Scytalidium* sp.	NI	HSV-1	ED_50_ = 2.2 μM	ND
halovir D [101]	peptide	*Scytalidium* sp.	NI	HSV-1	ED_50_ = 2.0 μM	ND
halovir E [101]	peptide	*Scytalidium* sp.	NI	HSV-1	ED_50_ = 3.1 μM	ND
balticolid [102]	polyketide	Ascomycetous fungus	driftwood	HSV-1	IC_50_ = 0.45 μM	ND
alternariol [106]	phenolic	*Pleospora tarda*	*Ephedra aphylla* endphyte	HSV-1	IC_50_ = 13.5 μM	ND
alternariol-(9)-methyl ether [106]	phenolic	*Pleospora tarda*	*E. aphylla* endophyte	HSV-1	IC_50_ = 21.3 μM	ND
oblongolide Z [107]	polyketide	*Phomopsis* sp. BCC 9789	*Musa acuminata* endophyte	HSV-1	IC_50_: 14 μM	ND
DHI [113]	phenolic	*Torrubiella tenuis* BCC 12732	Homoptera scale insect	HSV-1	IC_50_: 50 μg/mL	ND
cordyol C [114]	polyketide	*Cordyceps* sp. BCC 1861	Homoptera-cicada nymph	HSV-1	IC_50_: 1.3 μg/mL	ND
DTD [117]	polyketide	*Streptomyces hygroscopicus* 17997	GdmP mutant	HSV-1	IC_50_: 0.252 μgmol/L	ND
labyrinthopeptin A1/LabyA1 [118]	peptide	*Actinomadura namibiensis* DSM 6313	desert soil	HSV-1 and HSV-2	EC_50_ = 0.56 μM (HSV-1) and 0.32 μM (HSV-2)	entry
				HIV-1 and HIV-2	EC_50_ = 2.0 μM (HIV-1) and 1.9 μM (HIV-2)	entry
monogalactopyranose [120]	polyphenol	*Acremonium* sp. BCC 14080	palm leaf	HSV	IC_50_: 7.2 μM	ND
mellisol [121]	polyketide	*Xylaria mellisii* BCC 1005	NI	HSV	IC_50_: 10.5 μg/mL	ND
DOG [121]	polyketide	*Xylaria mellisii* BCC 1005	NI	HSV	IC_50_: 8.4 μg/mL	ND
spirostaphylotrichin X [126]	polyketide	*Cochliobolus lunatus* SCSIO41401	marine algae	H1N1 and H3N2	IC_50_: 1.6 μM (H1N1) and 4.1 μM (H3N2)	replication
cladosin C [127]	polyketide	*Cladosporium sphaerospermum* 2005-01-E3	deep sea sludge	H1N1	IC_50_: 276 μM	ND
abyssomicin Y [118]	polyketide	*Verrucosispora* sp. MS100137	deep sea sediment	H1N1	inhibition rate: 97.9%	ND
purpurquinone B [129]	polyketide	*Penicillium purpurogenum* JS03-21	acidic red soil	H1N1	IC_50_: 61.3 μM	ND
purpurquinone C [129]	polyketide	*Penicillium purpurogenum* JS03-22	acidic red soil	H1N1	IC_50_: 64 μM	ND
purpurester A [129]	polyketide	*Penicillium purpurogenum* JS03-23	acidic red soil	H1N1	IC_50_: 85.3 μM	ND
TAN-931 [129]	polyketide	*Penicillium purpurogenum* JS03-24	acidic red soil	H1N1	IC_50_: 58.6 μM	ND
pestalotiopsone B [130]	polyketide	*Diaporthe* sp. SCSIO 41011	*Rhizophora stylosa* mangrove endophte	H1N1 and H3N2	IC_50_: 2.56 μM (H1N1) and 6.76 μM (H3N2)	ND
pestalotiopsone F [130]	polyketide	*Diaporthe* sp. SCSIO 41012	*R. stylosa* mangrove endophte	H1N1 and H3N2	IC_50_: 21.8 μM (H1N1) and 6.17 μM (H3N2)	ND
DMXC [130]	polyketide	*Diaporthe* sp. SCSIO 41013	*R. stylosa* mangrove endophte	H1N1 and H3N2	IC_50_: 9.4 μM (H1N1) and 5.12 μM (H3N2)	ND
5-chloroisorotiorin [130]	polyketide	*Diaporthe* sp. SCSIO 41014	*R. stylosa* mangrove endophte	H1N1 and H3N2	IC_50_: 2.53 μM (H1N1) and 10.1 μM (H3N2)	ND
3-deoxo-4b-deoxypaxilline [131]	alkaloid	*Penicillium camemberti*	mangrove sediment	H1N1	IC_50_: 28.3 μM	ND
DCA [131]	alkaloid	*P. camemberti* OUCMDZ-1492	mangrove sediment	H1N1	IC_50_: 38.9 μM	ND
DPT [131]	alkaloid	*P. camemberti* OUCMDZ-1492	mangrove sediment	H1N1	IC_50_: 32.2 μM	ND
9,10-diisopentenylpaxilline	alkaloid	*P. camemberti* OUCMDZ-1492	mangrove sediment	H1N1	IC_50_: 73.3 μM	ND
TTD [131]	alkaloid	*P. camemberti* OUCMDZ-1492	mangrove sediment	H1N1	IC_50_: 34.1 μM	ND
emindole SB [131]	alkaloid	*P. camemberti* OUCMDZ-1492	mangrove sediment	H1N1	IC_50_: 26.2 μM	ND
21-isopentenylpaxilline [131]	alkaloid	*P. camemberti* OUCMDZ-1492	mangrove sediment	H1N1	IC_50_: 6.6 μM	ND
paspaline [131]	alkaloid	*P. camemberti* OUCMDZ-1492	mangrove sediment	H1N1	IC_50_: 77.9 μM	ND
paxilline [131]	alkaloid	*P. camemberti* OUCMDZ-1492	mangrove sediment	H1N1	IC_50_: 17.7 μM	ND
(14S)-oxoglyantrypine [132]	alkaloid	*Cladosporium* sp. PJX-41	mangrove sediment	H1N1	IC_50_: 85 μM	ND
norquinadoline A [132]	alkaloid	*Cladosporium* sp. PJX-42	mangrove sediment	H1N1	IC_50_: 82 μM	ND
deoxynortryptoquivaline [132]	alkaloid	*Cladosporium* sp. PJX-43	mangrove sediment	H1N1	IC_50_: 85 μM	ND
deoxytryptoquivaline [132]	alkaloid	*Cladosporium* sp. PJX-44	mangrove sediment	H1N1	IC_50_: 85 μM	ND
tryptoquivaline [132]	alkaloid	*Cladosporium* sp. PJX-45	mangrove sediment	H1N1	IC_50_: 89 μM	ND
quinadoline B [132]	alkaloid	*Cladosporium* sp. PJX-46	mangrove sediment	H1N1	IC_50_: 82 μM	ND
22-O-(N-Me-l-valyl)-21-epi-aflaquinolone B [138]	alkaloid	*Aspergillus* sp strain XS-2009	*Muricella abnormaliz* gorgonian	RSV	IC_50_: 0.042 μM	ND
aflaquinolone D [138]	alkaloid	*Aspergillus* sp strain XS-2009	*M. abnormaliz* gorgonian	RSV	IC_50_: 6.6 μM	ND
aurasperone A [152]	polyphenol	*Aspergillus niger* No.LC582533	*Phallusia nigra* tunicate	SARS-CoV-2	IC_50_: 12.25 μM	replication
neoechinulin A [153]	alkaloid	*Aspergillus fumigatus* MR2012	marine sediment	SARS-CoV-2	IC_50_: 0.47 μM	replication
aspulvinone D [154]	polyphenol	*Cladosporium* sp. 7951	*Paris polyphylla* endophyte	SARS-CoV-2	IC_50_: 10.3 μM	replication
aspulvinone M [154]	polyphenol	*Cladosporium* sp. 7951	*P. polyphylla* endophyte	SARS-CoV-2	IC_50_: 9.4 μM	replication
aspulvinone R [154]	polyphenol	*Cladosporium* sp. 7952	*P. polyphylla* endophyte	SARS-CoV-2	IC_50_: 7.7 μM	replication

Abbreviations: * ND: not yet described, * NI; no information, * DTM: 1,3-dihydro-4,5,6-trihydroxy-7-methylisobenzofuran, * THA: 1,2,4,5-tetrahydroxy-7-((2R)-2-hydroxypropyl) anthracene-9,10-dione, * MDMX: methyl 6,8-dihydroxy-3-methyl-9-oxo-9H-xanthene-1-carboxylate, * DHI: 6,8-dihydroxy-3-hydroxymethyl isocoumarin, * DOG: 1,8-dihydroxynaphthol 1-O-glucopyranoside, * DMXC: 3,8-dihydroxy-6-methyl-9-oxo-9H-xanthene-1-carboxylate, * TTD: (6S,7R,10E,14E)-16-(1H-indol-3-yl)-2,6,10,14-tetramethylhexadeca-2,10,14-triene-6,7-diol, * DTD: 4,5-dihydro-thiazinogeldanamycin, * DCA: 4a-demethylpaspaline-4a-carboxylic acid, * DPT: 4a-demethylpaspaline-3,4,4a-triol.

**Table 2:** Virus taxonomy, virus type, particle structure, and host receptor.

	HIV	HCV	HBV	Influenza Virus	HSV	RSV	SARS-CoV-2
**Taxonomy**							
Family	Retroviridae	Flaviridae	Hepadnaviridae	Orthomyxoviridae	Herpesviridae	Paramyxoviridae	Coronaviridae
Genus	Lentivirus	Hepacivirus	Orthohepadnavirus	Alphainfluenzavirus	Simplexvirus	Orthopneumovirus	Betacoronavirus
**Type**	positive-strand RNA	positive-strand RNA	partially double-stranded DNA	negative-strand RNA	double-stranded DNA	negative- strand RNA	positive-strand RNA
**Viral structure**							
Genome size	9.2 kb	±3 kB	3.2 kb	0.89–2.3 kb	125 kb	15.2 kb,	±29.9 kB
Core shape and diameter	cone-shaped and 145 nm	spherical and 40-80 nm	spherical or filamentous and 42 nm	spherical or pleomorphic and 80–120 nm	spherical and 155-240 nm	filamentous and 130 nm	spherical or ellipsoidal and 108 nm
Envelope glycoprotein	SU (gp120) and TM (gp41)	E1/E2 heterodimers, p7	LHBs, MHBs (preS1 and preS2) and SHBs	HA, NA	gD, gH-gL, gB, and additional gK, gC-gG, gE/gI, gN, gM, UL45	glycoprotein (G) and the fusion (F) glycoprotein	CoV envelope (E)
Non-structural protein	Gag-pol	NS2, NS3, NS4A, NS4B, NS5A, and NS5B	HBeAg and HBx	PA-X, PB1-F2, PB1-N40, PA-N155, PA-N182, M42 and NS3	-	NS1 and NS2	NSP 1 to NSP 16
**Host**							
Receptor (coreceptor)	CD4 receptor; CXCR4, and CCR5 coreceptors	CD81, Claudin 1, Occludin; LXRs	NTCP	SA	gD receptor: nectin-1, HVEM, 3-OS HS;NatasagB receptor: PILRα, MAG, NMHC-IIA	CX3CR1, nucleolin, EGFR, IGF1R, ICAM-1	ACE2
Attachment factor	DC-SIGN, L-SIGN	SR-B1, LDL	HSPG	-	HSPG	HSPG	L-SIGN

Abbreviations: SU, surface protein; TM, transmembrane glycoprotein; L in LHBs, large; M in MHBs, medium; S in SHBs, small; HA, hemagglutinin; NA, neuraminidase; gag-pol, gag (group antigen) and pol (polymerase); ACE2, angiotensin-converting enzyme 2; CCR5, cysteine-cysteine chemokine receptor type 5; CD, cluster of differentiation; CXCR4, cysteine-X-cysteine motif chemokine receptor 4; DC-SIGN, dendritic cell–specific intercellular adhesion molecule-3-grabbing non-integrin; HVEM, herpesvirus entry mediator; ICAM-1, intracellular adhesion molecule 1; L-SIGN, liver/lymph node-specific ICAM-1 grabbing non-integrin; LDLR, low-density lipoprotein receptor; SR-B1, scavenger receptor class A; NTCP, receptor sodium taurocholate co-transporting polypeptide, PILRα, paired immunoglobulin-like type 2 receptor α; MAG, myelin-associated glycoprotein, 3-OSHS, 3-O-sulfated heparan sulfate, CX3CR1, CX3C chemokine receptor 1; EGFR, epidermal growth factor receptor; IGF1R, insulin-like growth factor-1 receptor.
